# Supplementary material for: Impact of Amyloid Pathology in Mild Cognitive Impairment Subjects: The Longitudinal Cognition and Surface Morphometry Data
Source: Int J Mol Sci. 2022 Nov 23;23(23):14635. doi: 10.3390/ijms232314635 (PMC9738566; doi:10.3390/ijms232314635)
Supplement: Supplementary file 1 [file ijms-23-14635-s001.zip › ijms-1926831-supplementary.pdf]

Impact of Amyloid Pathology in Mild Cognitive Impairment Subjects: The Longitudinal Cognition and Surface Morphometry Data

Hsin-I Chang [1,†], Shih-Wei Hsu [2,†], Zih-Kai Kao [3], Chen-Chang Lee [2], Shu-Hua Huang [4], Ching-Heng Lin [5,6], Mu-N Liu [7,8,*], Chiung-Chih Chang [1,*]

Supplementary Content

Supplementary Result

Result S1. Longitudinal analysis: Cognitive decline (MMSE and CASI)

Supplementary Tables

Table S1. Mixed Effect Model Showing Interactions with longitudinal Mini-mental State Examinations
Table S2. Longitudinal cognitive ability screening instrument (CASI) total scores among groups
Table S3. Longitudinal short term memory scores among groups
Table S4. Memory pattern differences between Aβ+ and Aβ- groups
Table S5. Longitudinal language scores among groups
Table S6. Longitudinal verbal fluency scores among groups
Table S7. Longitudinal drawing scores among groups
Table S8. Longitudinal abstract thinking scores among groups
Table S9. Longitudinal long term memory scores among groups
Table S10. Longitudinal orientation scores among groups
Table S11. Longitudinal mental manipulation scores among groups
Table S12. Longitudinal attentional scores among groups

Supplementary Method

Method S1. MR image acquisition and processing
Method S2. Statistical analyses

Supplementary Result

Result S1. Longitudinal analysis: Cognitive decline (MMSE and CASI)

For MMSE (**Table S1**), both Aβ+ and Aβ- MCI groups had scored lower than controls. The MMSE decline was related to disease duration in Aβ+ but not in Aβ- MCI. Educational levels were not related to the decline in Aβ+ or Aβ- MCI. For CASI total scores (**Table S2**), both Aβ+ and Aβ- MCI groups had scored lower than controls, and the educational levels were significantly related to the total scores. Similarly, the decline of CASI was related to the disease duration in Aβ+ MCI but not in Aβ- MCI.

Based on the abovementioned result, we further explored the baseline to 5 years follow-up scores using the polynomial second order trendlines (with 95% Confidence intervals: **Figure 2**). Aβ+ MCI patients showed wider ranges of scores during the follow-ups, and the decline using the turning point function showed differences with Aβ- MCI or controls. For CASI STM and orientation subscores, the fall started from the baseline, while the others began to decline from year 1. The trendline for Aβ- MCI and controls, in contrast, are in parallel, although the scores of Aβ- MCI were all lower than the controls in MMSE, CASI total, and CASI subdomains.

Based on the memory performance differences in Aβ+ and Aβ- MCI at baseline, we further analyzed the STM subscore (**Table S3**), CVVLT delay recall (10minutes), and cue-correct score (**Table S4**). For STM, the results suggested lower scores in Aβ+ and Aβ- MCI compared with controls, but the decline related to disease duration was only found in Aβ+ MCI. In the 10-minute recalls, only Aβ+ MCI showed a lower score than controls, and the disease duration was also associated with the decline of 10-minute recalls. For cue-correct, only Aβ+ MCI showed a lower score than controls, and the disease duration was related to the decline in both Aβ+ MCI and Aβ- MCI.

In addition to STM, there were 8 cognitive subdomains in CASI. In the non-executive domains (**Table S5,S7,S9,S10**), both Aβ+ and Aβ- MCI groups had group effects on long term memory domains and orientation scores compared with the controls. In the executive domains (**Table S6,S8,S11,S12**), Aβ+ MCI showed no group effect but was significant for Aβ+ × disease duration interactions. The Aβ+ × disease duration effects were found in all 8 subdomains, while the Aβ- × disease duration effect was found in the orientation subdomains (**Table 2**).

Supplementary Tables

Table S1. Mixed effect model showing interactions with longitudinal Mini-Mental State Examinations (MMSE)

Parameter	B	Std. Error	95% Wald Confidence Interval		Hypothesis Test	
			Lower	Upper	Wald Chi-Square	Sig.
(Intercept)	24.943	1.4220	22.156	27.731	307.667	0.0001
Diagnosis						
**Aβ+**	-3.983	1.6443	-7.206	-0.761	5.869	0.015
**Aβ-**	-3.787	1.6449	-7.011	-0.563	5.301	0.021
Reference (age-matched controls)	0					
Education level	0.195	0.1319	-0.063	0.454	2.189	0.139
Aβ+ × Education	0.111	0.1673	-0.216	0.439	0.444	0.505
Aβ- × Education	0.148	0.1556	-0.157	0.453	0.905	0.341
Control × Education	0					
**Aβ+ × Disease duration**	-0.101	0.0145	-0.130	-0.073	48.871	0.0001
Aβ- × Disease duration	-0.029	0.0221	-0.072	0.014	1.700	0.192

Dependent variable: MMSE
Model: (intercept), diagnosis, education, diagnosis × education (years), diagnosis × duration (months)
Aβ-: patients without amyloid burden; Aβ+：patients with amyloid burden.

Table S2. Longitudinal Cognitive Ability Screening Instrument (CASI) total scores among groups

Parameter	B	Std. Error	95% Wald Confidence Interval		Hypothesis Test	
			Lower	Upper	Wald Chi-Square	Sig.
(Intercept)	78.686	3.3452	72.129	85.242	553.268	<0.0001
**Aβ+**	-10.023	3.9227	-17.711	-2.334	6.528	0.011
**Aβ-**	-13.335	3.9184	-21.014	-5.655	11.581	0.001
Reference (age-matched controls)	0					
**Education**	1.041	0.3422	0.371	1.712	9.260	0.002
Aβ+ × Education	0.257	0.4048	-0.537	1.050	0.402	0.526
Aβ- × Education	0.490	0.3976	-0.290	1.269	1.516	0.218
Control × Education	0					
**Aβ+** × **Disease duration**	-0.399	0.0333	-0.465	-0.334	143.801	<0.0001
Aβ- × Disease duration	-0.017	0.0226	-0.061	0.027	0.584	0.445

Dependent Variable: Total scores of CASI
Model: (intercept), diagnosis, education, diagnosis × education (years), diagnosis × duration (months)
Aβ-: patients without amyloid burden; Aβ+：patients with amyloid burden.

Table S3. Longitudinal short term memory scores among groups

Parameter	B	Std. Error	95% Wald Confidence Interval		Hypothesis Test	
			Lower	Upper	Wald Chi-Square	Sig.
(Intercept)	9.921	0.7773	8.397	11.444	162.894	<0.0001
**Aβ+**	-5.394	0.8985	-7.155	-3.633	36.034	<0.0001
**Aβ-**	-4.149	1.0318	-6.171	-2.127	16.168	<0.0001
Reference (age-matched controls)	0					
Education	-0.002	0.0846	-0.168	0.164	0.001	0.980
Aβ+ × Education	0.064	0.0976	-0.128	0.255	0.425	0.514
Aβ- × Education	0.167	0.1086	-0.046	0.380	2.361	0.124
Control × Education	0					
**Aβ+ × Disease duration**	-0.029	0.0053	-0.039	-0.019	30.042	<0.0001
Aβ- × Disease duration	0.000	0.0054	-0.010	0.011	0.004	0.949

Dependent Variable: short term memory scores of Cognitive Ability Screening Instrument (CASI)
Model: (intercept), diagnosis, education, diagnosis × education (years), diagnosis × duration (months)
Aβ-: patients without amyloid burden; Aβ+：patients with amyloid burden.

Table S4. Memory pattern differences between Aβ+ and Aβ- groups

Parameter	B	Std. Error	95% Wald Confidence Interval		Hypothesis Test	
			Lower	Upper	Wald Chi-Square	Sig.
**10-minute recalls (9)**						
**Aβ+**	-3.961	1.0567	-6.032	-1.890	14.051	<0.0001
Aβ-	-2.320	1.2468	-4.764	0.124	3.463	0.063
Reference (age-matched controls)	0					
**Aβ+ × Disease duration**	-0.022	0.0069	-0.036	-0.009	10.364	0.001
Aβ- × Disease duration	-0.029	0.0163	-0.061	0.004	3.045	0.081
**Cue Correct (9)**						
**Aβ+**	-3.633	1.0182	-5.628	-1.637	12.729	<0.0001
Aβ-	-2.182	1.2491	-4.630	0.266	3.051	0.081
Reference (age-matched controls)	0					
**Aβ+ × Disease duration**	-0.025	0.0058	-0.037	-0.014	19.247	<0.0001
Aβ- × Disease duration	-0.034	0.0095	-0.053	-0.016	13.068	<0.0001

Dependent Variable: 10-minute recalls or Cue Correct scores
Model: (intercept), diagnosis, diagnosis × duration (months)
Aβ-: patients without amyloid burden; Aβ+：patients with amyloid burden.

Table S5 Longitudinal language scores among groups

Parameter	B	Std. Error	95% Wald Confidence Interval		Hypothesis Test	
			Lower	Upper	Wald Chi-Square	Sig.
(Intercept)	8.141	0.5052	7.151	9.131	259.703	<0.0001
Aβ+	-0.088	0.5710	-1.207	1.031	0.024	0.877
Aβ-	0.014	0.5802	-1.123	1.151	0.001	0.981
Reference (age-matched controls)	0					
**Education**	0.142	0.0465	0.050	0.233	9.267	0.002
Aβ+ × Education	0.017	0.0540	-0.089	0.123	0.099	0.753
Aβ- × Education	-0.025	0.0513	-0.126	0.075	0.244	0.622
Control × Education	0					
**Aβ+ × Disease duration**	-0.038	0.0046	-0.046	-0.029	66.896	<0.0001
Aβ- × Disease duration	0.000	0.0023	-0.005	0.004	0.032	0.858

Dependent variable: language subdomain scores of Cognitive Ability Screening Instrument (CASI)
Model: (intercept), diagnosis, education, diagnosis × education (years), diagnosis × duration (months)
Aβ-: patients without amyloid burden; Aβ+：patients with amyloid burden.

Table S6. Longitudinal verbal fluency scores among groups

Parameter	B	Std. Error	95% Wald Confidence Interval		Hypothesis Test	
			Lower	Upper	Wald Chi-Square	Sig.
(Intercept)	5.424	0.6593	4.132	6.716	67.696	<0.0001
Aβ+	0.097	0.7862	-1.444	1.638	0.015	0.902
Aβ-	-1.494	0.8705	-3.200	0.213	2.944	0.086
Reference (age-matched controls)	0					
**Education**	0.172	0.0641	0.046	0.297	7.159	0.007
Aβ+ × Education	-0.085	0.0779	-0.238	0.067	1.201	0.273
Aβ- × Education	0.044	0.0869	-0.126	0.215	0.262	0.609
Control × Education	0					
**Aβ+ × Disease duration**	-0.028	0.0029	-0.034	-0.022	91.879	<0.0001
Aβ- × Disease duration	0.001	0.0051	-0.009	0.011	0.029	0.864

Dependent variable: verbal fluency subdomain scores of Cognitive Ability Screening Instrument (CASI)
Model: (intercept), diagnosis, education, diagnosis × education (years), diagnosis × duration (months)
Aβ-: patients without amyloid burden; Aβ+：patients with amyloid burden.

Table S7. Longitudinal drawing scores among groups

Parameter	B	Std. Error	95% Wald Confidence Interval		Hypothesis Test	
			Lower	Upper	Wald Chi-Square	Sig.
(Intercept)	8.761	0.3051	8.163	9.359	824.470	<0.0001
Aβ+	-0.738	0.4384	-1.598	0.121	2.838	0.092
Aβ-	-0.863	0.4640	-1.772	0.047	3.458	0.063
Reference (age-matched controls)	0					
**Education**	0.077	0.0378	0.003	0.151	4.153	0.042
Aβ+ × Education	-0.085	0.0779	-0.238	0.067	1.201	0.273
Aβ- × Education	0.044	0.0869	-0.126	0.215	0.262	0.609
Control × Education	0					
**Aβ+ × Disease duration**	-0.044	0.0039	-0.052	-0.037	127.742	<0.0001
Aβ- × Disease duration	0.002	0.0026	-0.003	0.007	0.560	0.454

Dependent variable: drawing subdomain scores of Cognitive Ability Screening Instrument (CASI)
Model: (intercept), diagnosis, education, diagnosis × education (years), diagnosis × duration (months)
Aβ-: patients without amyloid burden; Aβ+：patients with amyloid burden.

Table S8. Longitudinal abstract thinking scores among groups

Parameter	B	Std. Error	95% Wald Confidence Interval		Hypothesis Test	
			Lower	Upper	Wald Chi-Square	Sig.
(Intercept)	8.417	0.3716	7.689	9.146	513.185	<0.0001
Aβ+	-0.032	0.4442	-0.903	0.838	0.005	0.942
**Aβ-**	-1.051	0.4910	-2.013	-0.088	4.579	0.032
Reference (age-matched controls)	0					
**Education**	0.164	0.0391	0.087	0.240	17.544	<0.0001
Aβ+ × Education	-0.049	0.0466	-0.141	0.042	1.121	0.290
Aβ- × Education	-0.011	0.0496	-0.108	0.086	0.048	0.827
Control × Education	0					
**Aβ+ × Disease duration**	-0.046	0.0036	-0.053	-0.039	165.389	<0.0001
Aβ- × Disease duration	0.000	0.0028	-0.005	0.006	0.029	0.864

Dependent variable: abstract thinking subdomain scores of Cognitive Ability Screening Instrument (CASI)
Model: (intercept), diagnosis, education, diagnosis × education (years), diagnosis × duration (months)
Aβ-: patients without amyloid burden; Aβ+：patients with amyloid burden.

Table S9. Longitudinal long term memory scores among groups

Parameter	B	Std. Error	95% Wald Confidence Interval		Hypothesis Test	
			Lower	Upper	Wald Chi-Square	Sig.
(Intercept)	9.921	0.7773	8.397	11.444	162.894	<0.0001
**Aβ+**	-5.394	0.8985	-7.155	-3.633	36.034	<0.0001
**Aβ-**	-4.149	1.0318	-6.171	-2.127	16.168	<0.0001
Reference (age-matched controls)	0					
Education	-0.002	0.0846	-0.168	0.164	0.001	0.980
Aβ+ × Education	0.064	0.0976	-0.128	0.255	0.425	0.514
Aβ- × Education	0.167	0.1086	-0.046	0.380	2.361	0.124
Control × Education	0					
**Aβ+ × Disease duration**	-0.029	0.0053	-0.039	-0.019	30.042	<0.0001
Aβ- × Disease duration	0.000	0.0054	-0.010	0.011	0.004	0.949

Dependent variable: long term memory subdomain scores of Cognitive Ability Screening Instrument (CASI)
Model: (intercept), diagnosis, education, diagnosis × education (years), diagnosis × duration (months)
Aβ-: patients without amyloid burden; Aβ+：patients with amyloid burden.

Table S10. Longitudinal orientation scores among groups

Parameter	B	Std. Error	95% Wald Confidence Interval		Hypothesis Test	
			Lower	Upper	Wald Chi-Square	Sig.
(Intercept)	16.856	0.6227	15.635	18.076	732.690	<0.0001
**Aβ+**	-4.387	0.8703	-6.092	-2.681	25.408	<0.0001
**Aβ-**	-3.825	1.0493	-5.882	-1.768	13.287	<0.0001
Reference (age-matched controls)	0					
Education	0.059	0.0715	-0.082	0.199	0.671	0.413
Aβ+ × Education	0.156	0.0893	-0.019	0.331	3.065	0.080
**Aβ- × Education**	0.204	0.1010	0.007	0.402	4.101	0.043
Control × Education	0					
**Aβ+ × Disease duration**	-0.082	0.0066	-0.095	-0.069	152.954	<0.0001
**Aβ- × Disease duration**	-0.018	0.0067	-0.031	-0.005	7.497	0.006

Dependent variable: orientation subdomain scores of Cognitive Ability Screening Instrument (CASI)
Model: (intercept), diagnosis, education, diagnosis × education (years), diagnosis × duration (months)
Aβ-: patients without amyloid burden; Aβ+：patients with amyloid burden.

Table S11. Longitudinal mental manipulation scores among groups

Parameter	B	Std. Error	95% Wald Confidence Interval		Hypothesis Test	
			Lower	Upper	Wald Chi-Square	Sig.
(Intercept)	6.507	0.7760	4.986	8.028	70.315	<0.0001
Aβ+	-0.948	0.8335	-2.582	0.686	1.294	0.255
Aβ-	-1.765	0.9083	-3.545	0.015	3.777	0.052
Reference (age-matched controls)	0					
**Education**	0.183	0.0613	0.062	0.303	8.878	0.003
Aβ+ × Education	0.073	0.0676	-0.059	0.206	1.175	0.278
Aβ- × Education	0.089	0.0745	-0.057	0.235	1.432	0.231
Control × Education	0					
**Aβ+ × Disease duration**	-0.042	0.0029	-0.047	-0.036	212.683	<0.0001
Aβ- × Disease duration	0.004	0.0028	-0.002	0.009	1.907	0.167

Dependent variable: mental manipulation subdomain scores of Cognitive Ability Screening Instrument (CASI)
Model: (intercept), diagnosis, education, diagnosis × education (years), diagnosis × duration (months)
Aβ-: patients without amyloid burden; Aβ+：patients with amyloid burden.

Table S12. Longitudinal attentional scores among groups

Parameter	B	Std. Error	95% Wald Confidence Interval		Hypothesis Test	
			Lower	Upper	Wald Chi-Square	Sig.
(Intercept)	6.311	0.2110	5.898	6.725	894.292	<0.0001
Aβ+	0.299	0.2657	-0.221	0.820	1.269	0.260
Aβ-	-0.031	0.2964	-0.613	0.550	0.011	0.915
Reference (age-matched controls)	0					
**Education**	0.088	0.0167	0.055	0.121	27.809	<0.0001
Aβ+ × Education	-0.022	0.0248	-0.071	0.026	0.807	0.369
Aβ- × Education	-0.006	0.0280	-0.061	0.049	0.049	0.825
Control × Education	0					
**Aβ+ × Disease duration**	-0.027	0.0028	-0.032	-0.021	90.423	<0.0001
Aβ- × Disease duration	-0.001	0.0020	-0.005	0.003	0.222	0.637

Dependent variable: attention subdomain scores of Cognitive Ability Screening Instrument (CASI)
Model: (intercept), diagnosis, education, diagnosis × education (years), diagnosis × duration (months)
Aβ-: patients without amyloid burden; Aβ+：patients with amyloid burden.

Supplementary Method

Method S1: MRI Data acquisition and processing

MR image acquisition

Each subject received a T2-weighted MRI scan to confirm the absence of organic lesions in the brain. White matter hyperintensities of Fazekas scale >2 were excluded. 3DT1 MR images were obtained using a 3T GE Discovery 750 (GE Medical Systems, Milwaukee, WI, USA) and was acquired using a T1-weighted, inversion-recovery-prepared, three-dimensional, gradient-recalled acquisition in a steady-state sequence [repetition time (TR) = 12.24 msec; echo time (TE) = 5.18 msec; field of view (FOV) = 256 × 256; matrix size= 256 × 256; number of excitations (NEX) = 1; inversion time (TI) = 450 msec; flip angle = 15] with a 1-mm slice sagittal thickness with a resolution of 0.5 × 0.5 × 1 mm^3.

Preprocessing of baseline and longitudinal imaging data

All MRI scans were processed on the same workstation (Macintosh iMac Pro 2017, MacOS Catalina, version 10.15.16) using FreeSurfer image analysis suite v7.1.1 (http://surfer.nmr.mgh.harvard.edu). Cortical reconstruction and volumetric segmentation of MRI were estimated using the automated processing stream (recon-all). The preprocessing procedures carried out by FreeSurfer included removing nonbrain data, intensity normalization, skull-stripping, segmentation of cerebrospinal fluid, white matter, and gray matter. Surface-based registration was used to align cortical folds with a brain template, and then volumetric registration was used to reconstruct images. Following the reconstruction process, every MR image was visually inspected slice by slice in horizontal, coronal, and sagittal views using Freeview to ensure accuracy of registration, skull stripping, segmentation, and cortical surface reconstruction. Manual corrections were performed when tissue misclassifications were encountered. The distance between the white and pial surfaces at a vertex is defined as the cortical thickness at that vertex [49]. We chose the gray matter thickness as the dependent variable in this study.

For the longitudinal process, the unbiased within-subject template was created using robust, inverse consistent registration among the time points [50]. The use of the within-subject template with the preprocessing steps of skull stripping, Talairach transformations, atlas registration, spherical surface maps and parcellations significantly increase longitudinal streams' reliability and statistical power. Images were then smoothed using a Gaussian kernel across the surface with a full width at

half maximum (FWHM) of 10 mm. The cortical thickness was calculated as the closest distance from the grey/white matter boundary to the gray matter/CSF boundary of each vertex.

Method S2. Statistical analyses

Cross-sectional cognitive data for the differentiation of 3 groups were analyzed using analyses of variance followed by Bonferroni corrections and Chi-Square for categorical data. For the time effect, we calculate intervals (months) between disease onset and cognitive test date. For the control group, the disease duration was defined as the months with the baseline visit. For longitudinal cognitive data, we used LME models with group, time and an interaction between group and time to assess the cognitive changes. Covariates adjusted for the analysis include baseline cognitive test score, age, education. The Bonferroni correction was used to correct for multiple comparisons. The subject specific random intercept and slope for time were included in the LME model to account for correlations among repeated measures of the cognitive outcomes. R version 3.0.1 (R Core Team, 2013; URL http://www.R-project.org/) and RStudio (Rstudio, 2012; URL http://www.rstudio.org/) were used for the analysis and R package FindMinIC v1.6 (http://CRAN.R-project.org/ package=FindMinIC) for LME model analysis. For the longitudinal cognitive test plot, we used python3.8.5 and the second-degree polynomial fit to generate the longitudinal cognitive decline trajectory. For the decliner, a turning time point is defined as a function change from increasing to decreasing or vice-versa.

For MRI analysis, the LME model was performed with MATLAB 2019b (The Mathworks Inc., Natick, MA, USA) to test the relationships of cortical thickness with variables including group, disease duration (months), *APOE* ε4 effect, and cognitive scale. The model considered cortical measurements as a dependent variable and age, gender, education year, and estimated total intracranial volume (eTIV) as a nuisance covariate. The diagnostic groups, disease duration of each group, and interaction between diagnostic group and time were modeled. Within Aβ+ or Aβ- group, we further divided them into *APOE* ε4 carrier and non-carrier states to model the cross-sectional and longitudinal effect on cortical thickness. Meanwhile, the cognitive relationships with cortical thickness were constructed using correlation analysis within each group to explore the possible atrophy topography. Almost every MRI analysis involves thousands of simultaneous significance tests on the discrete region in the brain. With cluster-wise correction computed with parametric Gaussian-based simulations to compute the false positive rate 0.05, we used a vertex wise threshold of 3.0 [51].

Reference

49. Fischl, B.; Dale, A. M. Measuring the thickness of the human cerebral cortex from magnetic resonance images. *Proc Natl Acad Sci U S A* **2000,** *97,* 11050-5.
50. Reuter, M.; Schmansky, N. J.; Rosas, H. D.; Fischl, B. Within-subject template estimation for unbiased longitudinal image analysis. *Neuroimage* **2012,** *61,* 1402-18.
51. Greve, D. N.; Fischl, B. False positive rates in surface-based anatomical analysis. *Neuroimage* **2018,** *171,* 6-14
